# Hypereosinophilia‐related liver pseudotumor with elevated interleukin‐5 levels preceding T‐cell lymphoma

**DOI:** 10.1002/jgh3.12253

**Published:** 2019-09-06

**Authors:** Atsunori Tsuchiya, Tomoyuki Tanaka, Yasuhiko Shibasaki, Shuji Terai

**Affiliations:** ^1^ Division of Gastroenterology and Hepatology, Graduate School of Medical and Dental Sciences Niigata University Niigata Japan; ^2^ Department of Hematology, Endocrinology and Metabolism, Faculty of Medicine Niigata University Niigata Japan

**Keywords:** hypereosinophilia, interleukin‐5, malignant lymphoma, multiple liver tumors, ultrasonography

## Abstract

A 61‐year‐old woman with hypereosinophilia and elevated interleukin (IL)‐5 level was admitted to our hospital after detection of multiple liver tumors. Liver biopsy demonstrated that the tumor consisted of scar tissue with remnants of eosinophilic infiltration, suggesting that it had formed by massive eosinophilic infiltration. The hypereosinophilia was treated mainly by prednisolone, and thereafter, the liver tumors disappeared. However, 10 months postadmission, CD4+ T‐cell lymphoma, which can produce IL‐5, was detected in the nasopharynx and oropharynx. Therefore, we believe that this is a rare case of hypereosinophilia‐related liver pseudotumor induced by presumed by IL‐5 elevation.

## Graphical abstract

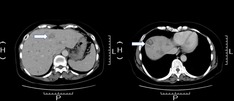

Multiple liver tumors due to hypereosinophilia caused by interleukin (IL)‐5 disappeared mainly on steroid therapy. IL‐5 elevation that induced hypereosinophilia was due to the potent hidden T‐cell malignant lymphoma.

## Introduction

Hypereosinophilia can lead to pseudotumors in the liver, most of which are treated by prednisolone.^1^ We report a rare case of hypereosinophilia‐related liver pseudotumor presumed by the elevation of interleukin (IL)‐5, which was produced by T‐cell lymphoma detected 10 months after detection of the pseudotumor in the liver.

## Case report

**Figure 1:**
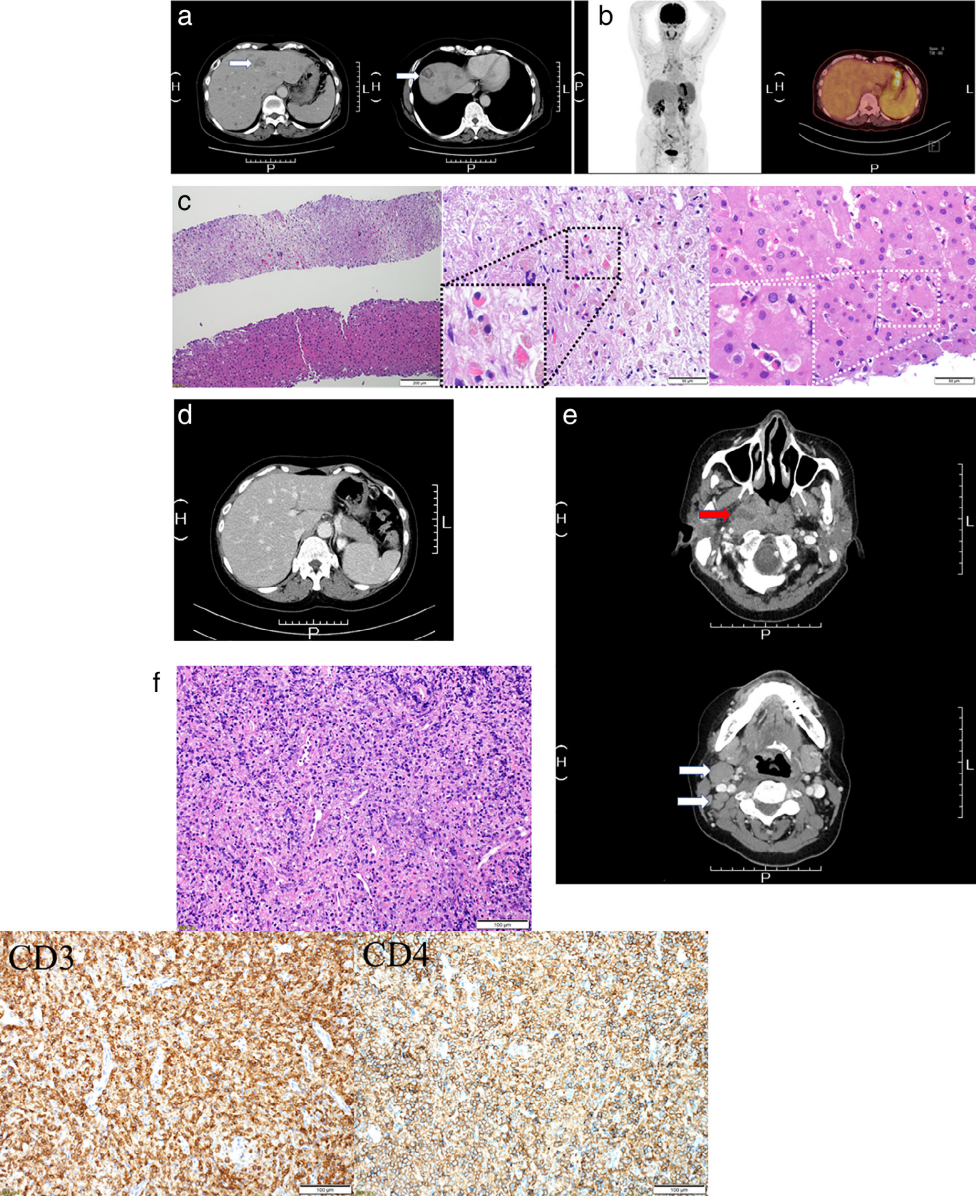
Computed tomography (CT) shows multiple hypovascular tumors (a; white arrows). Positron emission tomography‐CT could not detect primary tumor (b). Liver biopsy showed that the tumor consisted of scar tissue with remnants of eosinophil infiltration (c). Multiple liver tumors disappear after treatment (d). Ten months later, a hypovascular tumor in the nasopharynx and oropharynx (red arrow) and swelling of the surrounding lymph nodes (white arrows) appeared (e). Pathological analysis showed that the tumor was a T‐cell malignant lymphoma (f).

A 61‐year‐old woman was admitted to our hospital after multiple liver tumors were detected using computed tomography (CT) (Fig. 1a; white arrows), with chief complaints of continuous general fatigue and itching of 1 month's duration. Her regular health check‐ups during the preceding year were normal. A CT performed at another hospital showed that the liver tumors were hypovascular and were suspected to be metastatic liver tumors (Fig. 1a; white arrows). Therefore, upper gastrointestinal endoscopy, colonoscopy, and positron emission tomography‐CT (PET‐CT) were performed, but no primary lesions were detected (Fig. 1b). On admission, her laboratory data showed hypereosinophilia, a low platelet count, abnormal liver and biliary enzymes, and abnormalities of several tumor and inflammatory markers. Her white blood cell (WBC) count was 82 480/μL (normal range, 3300–8600/μL), percentage of eosinophils in WBCs was 83.1%, platelet count was 6.5 (normal range, 15.8–34.8 × 10^4^/μL), alanine aminotransferase was 43 IU/L (normal range, 8–42 IU/L), alkaline phosphatase was 2200 U/L (normal range, 106–322 U/L), and γ‐glutamyl transpeptidase was 190 IU/L (normal range, 13–64 IU/L). Carcinoembryonic antigen, carbohydrate antigen 19–9, α‐fetoprotein, and des‐gamma‐carboxy prothrombin were within the normal range; however, IL‐5 (55.4 pg/mL; normal range, <3.9 pg/mL), IgG4 (36.2; normal range, 11–121 mg/dL), and IL‐2 receptor (5625; normal range, 122–496 U/mL) levels were markedly elevated. After admission, ultrasonography (US) was performed to guide the liver biopsy; however, no tumors could be detected by conventional US.

Liver tumor‐like lesions could be seen as defects in the Kupffer phase of contrast‐enhanced US performed using Sonazid suspension (Daiichi Sankyo, Tokyo, Japan). Liver biopsy using a 21‐gauge needle showed that the tumor consisted of scar tissue with remnants of eosinophil infiltration. Eosinophil infiltration was also observed in the parenchyma surrounding the lesion (Fig. 1c). These histological findings and hypereosinophilia from laboratory data suggested that the tumor was formed by massive eosinophil infiltration or was due to the hypercoagulable state caused by the hypereosinophilia. Consequently, we interpreted the eosinophilic liver mass to be a manifestation of hypereosinophilic syndrome. After the liver biopsy, hypereosinophilia was treated by oral administration of prednisolone at an initial dose of 60 mg/day.^1^ Two weeks later, a follow‐up contrast‐enhanced CT confirmed that almost all the previously detected tumors had disappeared (Fig. 1d). The prednisolone was gradually tapered, during which 100–200 mg/day of imatinib mesylate and 500 mg/day or per 2 days of hydroxycarbamide were added. Ten months after the admission, she developed a sore throat. Contrast‐enhanced CT showed a hypovascular tumor in the nasopharynx and oropharynx (Fig. 1e; red arrow) and swelling of the surrounding lymph nodes (Fig. 1e; white arrows). Pathological analysis showed that the tumor was a malignant lymphoma: a nodal peripheral T‐cell lymphoma with the T follicular helper (TFH) phenotype. Immunohistochemistry analysis demonstrated that CD3, CD4 (Fig. 1f), PD‐1, and BCL‐6 were positive, while CD10 and CXCL13 were negative. Rearrangement of the T‐cell receptor β‐chain Cβ lesion was detected.

## Discussion

Hypereosinophilia can be caused by drugs, parasitosis, autoimmune diseases (Churg‐Strauss syndrome, Wegener granulomatosis), allergic inflammation to a specific antigen, and neoplastic hematopoietic disorders. In this case, the malignant lymphoma was not detected in the biopsy samples, CT, and PET‐CT performed during the first admission. However, it was readily apparent 10 months later. Looking back to the first admission, we could see that IL‐5, which is produced by CD4+ T cells and induces eosinophil proliferation, differentiation, and migration, was increased.^2^, ^3^ Although we could not detect abnormal T‐cells on the CT or blood test during the first admission, they were potentially present.
